# Modernizing Vaccination Data System: Design, Development, and Deployment of a Digital Vaccination Registry in Liberia, 2023–2025

**DOI:** 10.3390/vaccines14040323

**Published:** 2026-04-04

**Authors:** Olorunsogo Bidemi Adeoye, Dieula Delissaint Tchoualeu, Patrick K. Konwloh, Halima Abdu, Calvin Coleman, Abizeyimana Aime Theophile, Anthony Lucene Fortune, Yuah Nemah, Carl Kinkade, Oluwasegun Joel Adegoke, Eugene Lam, Denise Giles, Rachel T. Idowu

**Affiliations:** 1 Division of Global Health Protection, United States Centers for Disease Control and Prevention, Atlanta, GA 30329, USA; 2 Ministry of Health, Republic of Liberia Congo Town, Tubman Blvd, Monrovia 1000, Liberia; 3 United Nations Children’s Fund (UNICEF), Liberia Country Office, 4th Floor, One UN House, Pan African Plaza, 1st Street, Sinkor, Monrovia 1000, Liberia; 4 Global Immunization Division, United States Centers for Disease Control and Prevention, Atlanta, GA 30329, USA

**Keywords:** electronic immunization registry, District Health Information System (DHIS2), Liberia, routine immunization, digital health, collaborative requirements development methodology

## Abstract

Background: Liberia modernized vaccination data systems in 2023–2025 by piloting a District Health Information System (DHIS2)-based Digital Vaccination Registry (Electronic Immunization Registry, EIR) to address the limitations of paper-based workflows and of a proprietary COVID-19 electronic platform (offline gaps, lack of unique identifiers, performance issues and cost). Objective: To assess a pilot platform by evaluating training, registry use and device management, utility for routine immunization, vaccine logistics and Adverse Events Following Immunization (AEFI) data, and routine immunization data quality in the DHIS2 mobile application compared with paper registers. Methods: Using the Public Health Informatics Institute’s Collaborative Requirements Development Methodology, stakeholders defined requirements, trained users and implemented a pilot. Mixed methods were used; a mini data audit was performed, and qualitative data were collected across 19 facilities in Montserrado, Gbarpolu and Grand Bassa. Seventy-eight health workers were trained to use the DHIS2 mobile application. Results: The future state design replaces paper aggregation steps with real-time mobile entry to a national registry and dashboard. Dual entry persisted during high-volume periods. The mini data audit found discrepancies between facility paper registers and DHIS2-EIR entries for child enrollment data and, Bacillus Calmette Guérin and Diphtheria–Pertussis–Tetanus dose administration records Participants attributed these discrepancies to internet and device problems and challenges navigating the system. Participants requested a training manual, improved connectivity at point of service, integration with supportive supervision, additional staff and system features (field to record hospital number, automated next visit date, and vaccination status prompts). Conclusions: Lessons from the pilot will inform country-wide implementation, including planned linkage with electronic birth and death registration to enable a unique child identifier and reduce manual errors and delays.

## 1. Introduction

Routine Immunization (RI), provided in most countries, is the most strategic component of primary healthcare aimed at preventing diseases [1]. Accurate immunization information is essential for immunization managers to track and improve program performance and ultimately prevent morbidity and mortality [2,3]. The quality of the data used for healthcare decision-making has direct implications on people’s lives and well-being [4,5]. Timely and high-quality disease surveillance and vaccination data are necessary to inform sound decision-making and manage effective health programs [6,7]. A robust system for collecting, reporting, and archiving health services data is essential for providing accurate and timely information to support the effective planning, monitoring, and evaluation of service delivery.

Telehealth and digital tools are increasingly recognized as transformative solutions for expanding healthcare access in rural and underserved regions globally [8]. The World Health Organization (WHO) emphasizes the critical role these technologies play in achieving Universal Health Coverage (UHC) [9]. A study highlights their growing use in sub-Saharan Africa, especially in healthcare delivery, worker support, and surveillance [10]. In many low- and middle-income countries (LMICs), health facility vaccination information has traditionally been captured through paper-based tools at the point of service delivery [11,12,13]. However, as many LMICs are undergoing a digital transformation of their health systems, an opportunity exists to transition to digital health solutions for managing vaccination data [12,14]. Further, the global proliferation of low-cost mobile technology has led to a gradual shift towards digital data collection systems, and mobile health (mHealth) solutions are becoming increasingly common in the developed world as well as in LMICs [15]. The expansion of broadband connectivity has significantly facilitated the growth of mHealth initiatives in LMICs. This increased access to reliable internet has allowed for the implementation of diverse mHealth applications, reaching previously underserved populations [16].

The Digital Vaccination Registry, also known as the Electronic Immunization Registry (EIR), is a confidential, computerized, routine system used to capture, store, access, and share individual-level, longitudinal health information on vaccination that can provide powerful solutions to address multiple deficiencies in LMIC paper-based immunization systems [15,17,18]. For example, EIRs have been shown to strengthen immunization practices in LMICs by saving health workers’ time with automated reporting, improving data quality and use, and monitoring vaccine stock-out, ultimately providing data insights to identify the most high-risk children. An added benefit is the generation of reports to inform local and national policies [13,15,19,20,21,22,23,24,25,26,27]. A 2021 report found that EIRs had been piloted or implemented in more than 50 LMICs [19,28].

One of the most widely adopted platforms to support digital health information systems, including EIRs in LMICs, is the District Health Information System version 2 (DHIS2). DHIS2 is open-source software developed in a collaboration between the Health Information System Program (HISP) at the University of Oslo (UiO) and the global HISP network [29]. More than 80 countries worldwide and approximately 3.2 billion people use DHIS2 for collecting and analyzing health data [30]. The Digital Vaccination Registry (e-Registry) data package on DHIS2 was developed in 2017 to address the needs of countries and partners to improve the timeliness and accuracy of data from RI [31,32]. The Government of Vietnam, Tanzania and Zambia respectively, began designing and developing EIRs in 2010. These countries were among the earliest LMIC EIR adopters [28]. The DHIS2 e-Registry package provides templates and other resources for RI managers.

In Liberia, vaccination data are manually captured using paper-based tools at the point of service delivery and later aggregated for digital entry into the national DHIS2-based health management information system. During the design and launch of the national COVID-19 vaccination program, a proprietary digital platform was deployed but faced technical limitations including a lack of offline data capture, an inability to uniquely identify records, and poor performance of the app, contributing to backlogs and duplicated records. These limitations, alongside the need to reduce the fragmentation of digital tools and data silos, informed the decision to design and deploy a DHIS2-based Digital Vaccination Registry.

The objective of this study was to assess the three-county pilot of Liberia’s DHIS2-based Digital Vaccination Registry by evaluating training, registry usage and device management, utility for routine immunization, vaccine logistics and AEFI data management, and routine immunization data quality reported through the DHIS2 mobile application compared with paper-based registers.

## 2. Country Context

To support Liberia’s national COVID-19 vaccination program, the Liberia Ministry of Health (MoH) and National Public Health Institute of Liberia (NPHIL), in collaboration with other partner organizations, opted to digitize COVID-19 vaccination administration data for the first time. The Government of Liberia (GoL) initially deployed a proprietary commercial digital platform, anticipating eventual large-scale country-wide digitization. However, the platform soon faced an array of technical issues, ranging from a lack of offline data-capturing features (indispensable in a country with inconsistent internet connectivity), an inability to uniquely identify records, and poor performance of the mobile application used for reporting to the national-level Health Information System (HIS) Officers by the user. Consequently, large data entry backlogs, the duplication of immunization records, delayed processing of records, high recurring maintenance costs, and overall non-compliance with global principles of digital development quickly marred the utility of this platform.

Additionally, non-negotiable proprietary parameters limited the GoL’s leverage to augment existing DHIS2 infrastructure with the features of the commercial platform (e.g., equipment, personnel, funding, interoperability). This limited the utility of the commercial platform for other immunization program areas [e.g., disease surveillance, Adverse Events Following Immunization (AEFI), and vaccine stock management], thereby deepening the fragmentation of digital tools and data silos in Liberia’s health system.

Therefore, the MoH, with funding, technical leadership and oversight from the United States (U.S.) Centers for Disease Control and Prevention (CDC), United Nations Children’s Emergency Fund (UNICEF), and the West and Central Africa node of HISP, led the design, development, and deployment of a DHIS2-sourced digital vaccination registry to modernize the vaccination data system in Liberia. Through the authors’ firsthand experiences, we report on stakeholder engagement, system assessment, design, development, deployment, and pilot implementation in Liberia from 2023 to 2025.

## 3. Methods

**Figure 1:**
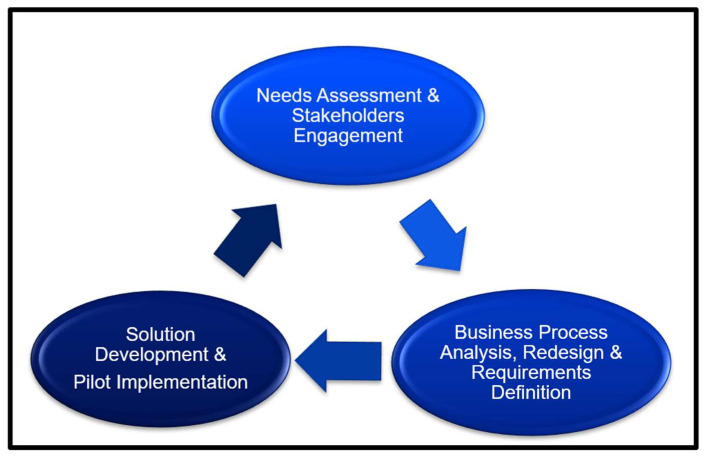
Adaptation of the Public Health Informatics Institute’s Collaborative Requirements Development Methodology (CRDM) for the Liberia Digital Vaccination Registry, showing the three steps applied: needs assessment; business process analysis, requirements definition, and vendor analysis; and solution implementation.

This approach was framed by the Public Health Informatics Institute’s (PHII) Collaborative Requirements Development Methodology (CRDM), which includes a validated four-step framework (needs assessment; business process analysis and requirements definition; vendor analysis; solution implementation) [33]. The phases and steps of the framework were adapted for the Liberian context to ensure a systematic approach to development using validated steps and tools, and also to align the concept development and implementation activities with recognized practices (Figure 1).

Stakeholders’ engagement the needs assessment were conducted using mixed methods that included key informant interviews, focus group discussions, system demonstration sessions, system inventory using a standard checklist, site visits for observation, and workshops with technical team members responsible for HIS management and the RI program at the national level [34]. The stakeholder group included the MoH teams (Health Information System, Monitoring and Evaluation, and RI), U.S. CDC, UNICEF, WHO, selected sub-national leaders, and healthcare workers.

Key informant interviews were conducted with 15 stakeholders at national and sub-national levels.

A business process analysis was carried out, and requirements were defined to describe the immunization program’s current process and data flow and to document bottlenecks from the user’s perspective across different levels. The future state design was also documented, describing areas of optimization and technologies that could add more value to the data life cycle from the user’s perspective (vaccinators, data entry clerks, county district and national data managers,) at different levels of the RI program.

This pilot of the digital solution was preceded by system selection and iterative development sessions that allowed stakeholders to agree on the final technological solution with the most value for adoption and use within the Liberian context. This involved the development and testing of the digital vaccination registry. All the technological solutions under consideration were reviewed for alignment with the requirements of the vaccination registry defined by the stakeholders. Technical leaders from MoH and NPHIL agreed to launch a digital vaccine registry within DHIS2. This met the largest number of the requirements described by the stakeholders.

Next, training opportunities were provided for pilot participants. Training objectives were to equip health workers (vaccinators, certified midwives, data officers, and monitoring and evaluation officers) in a sub-set of Liberia’s public facilities to use the EIR mobile application for timely and accurate entry, submission and the overall management of vaccination, vaccine logistics, and AEFI data.

For effective training and eventual successful implementation, the U.S. CDC and representatives for HISP West and Central Africa provided technical advice at all levels. Also, MoH and NPHIL subject matter experts (SMEs) served as facilitators. All facilitators were trained during the National Training of Trainers and then the training was extended to health workers at facilities within the pilot counties.

The objectives of this assessment were to quantitatively and qualitatively assess digital vaccination registry implementation training, as well as vaccination registry usage and individual user device management; examine the utility of the digital vaccination registry for RI, vaccine logistics and AEFI data management; and assess RI data quality (e.g., Bacillus Calmette-Guérin, Diphtheria–Tetanus–Pertussis, and child enrollment data) reported through the DHIS2 mobile application compared to paper-based registers (decentralized and national referral health facility DHIS2 registers).

Additionally, a complementary cross-sectional evaluation was conducted at 19 health facilities across three counties (Montserrado, Gbarpolu, and Grand Bassa). Facilities and counties were purposively selected to test feasibility, usability, and adaptability across high-, medium-, and low-volume settings and across hard-to-reach and easy-to-reach areas; therefore, evaluation findings may not be representative of all facilities or counties. The target population included all health workers trained on the Digital Vaccination Registry across the three counties. Quantitative (mini-data audit) and qualitative (focus group discussions and in-depth interviews) methods were used for data collection, along with key informant interviews.

Quantitative analysis using a mini data-audit instrument compared routine immunization totals recorded in facility paper registers with totals recorded in the DHIS2-EIR instance for the same variables and months; discrepancy was assessed as the point difference between sources and summarized as “percent discrepancy.” Qualitative data from focus group discussions and interviews were coded into short words or phrases summarizing segments of text and then grouped into themes and sub-themes for interpretation.

## 4. Outcomes

The core technical team from MoH, NPHIL, UNICEF, and the U.S. CDC finalized the implementation work plan, budget, training and meeting schedule, and provided overall oversight of the project. Fifteen stakeholders were interviewed at different levels. Six needs assessment sessions and four key informant sessions were conducted with the MoH Coordinator for Health Information Management and Research, the Director of Health Information System, the Director for Information Communication Technology, and the RI Manager. The sub-national needs assessment was conducted during consultative site visits at the health facilities. Following different rounds of technical considerations to review and understand the country’s requirements, DHIS2 was agreed upon as the platform of choice for the digital vaccination registry. The technical team recommended that Liberia digital vaccination registry be established using existing DHIS2 architecture and resources.

### 4.1. Current State

**Figure 2:**
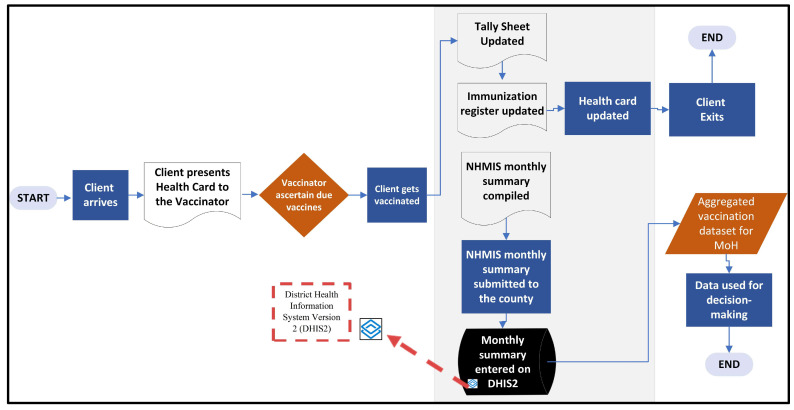
Current state of process and data flows of the Liberia routine immunization program. NHMIS—National Health Management Information System.

Figure 2 depicts the current process and data flows of the immunization program in Liberia, highlighting various paper forms and registers utilized for the collection and overall management of immunization data.

Vaccination data are generated at the point of service delivery. Different forms and registers (child health card, tally sheet, child immunization register, health facility monthly summary form, etc.) are used for data collection and aggregate reporting. Monthly datasets across all health facilities offering vaccination services are compiled by designated personnel and aggregated at the county level. Data officers then enter the aggregated datasets into Liberia’s DHIS2-based health management information system (HMIS) managed by the MoH (Health Information System Department and the Information, Communication and Technology Department). Reports on Liberia’s national immunization profile and program performance are provided to Liberia’s policymakers, donor and partner organizations, and all other relevant stakeholders for programmatic and public health decision-making.

### 4.2. Future State

**Figure 3:**
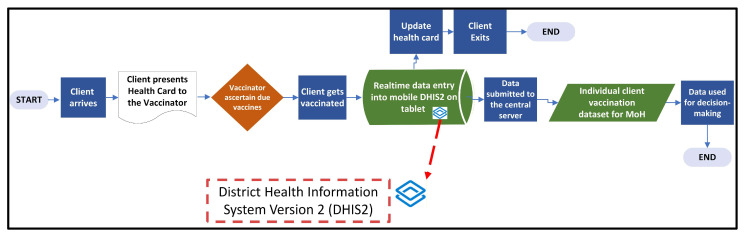
Desired future state of process and data flow design for vaccination data management in Liberia.

Figure 3 depicts the desired future state of the process and data flow that was agreed to by all stakeholders [MoH (Health Information System, Monitoring and Evaluation, and RI), U.S. CDC, UNICEF, WHO, selected sub-national leaders, and selected healthcare workers]. The future state design was informed by the needs of technical and program staff, as well as the need for quicker action by public health decision-makers.

**Figure 4:**
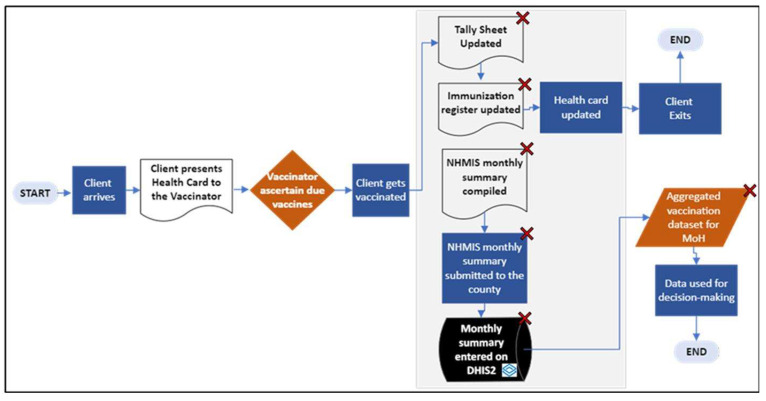
Areas (processes) marked with red “X” are identified for elimination to ensure the overall optimization of the system.

During this phase, the requirements of the proposed future state were collected, documented, validated, and approved by all stakeholders. Data collection processes and related paper forms marked to be eliminated as depicted in Figure 4 were identified (“Tally Sheet Updated,” “Immunization register updated,” “NHMIS monthly summary compiled,” “NHMIS monthly summary submitted to the county,” “Monthly summary entered on DHIS2,” and “Aggregated vaccination dataset for MoH”). It was anticipated that those processes would become redundant once the use of mobile devices for immunization data collection and transmission from the health facilities to national level was introduced.

In the future state, real-time data entry will occur using the mobile DHIS2 applications installed on government-furnished mobile devices to ensure digitized transmission into the national registry, where the data will populate selected immunization indicators on a national dashboard. This represents a significant advantage over the current paper-based methods, where data is only submitted monthly in aggregate format, limiting policymaker access to real-time data. Using a mobile device not only ensures the smooth transmission of immunization records but also facilitates easy, simultaneous access to individual child records at the facility level and above the facility level (via the mobile devices and the national dashboard, respectively). Additionally, use of mobile transmission automates calculations of relevant indicators, thus shortening the time required for analysis and decision-making.

### 4.3. Pilot Implementation

Seventy-eight participants were trained (28 Montserrado, 23 Gbarpolu, and 27 Grand Bassa). County-level training was conducted over three days. These training courses officially launched the EIR pilot implementation phase. At the conclusion of the training, each participant received a donor-supplied digital device to commence data collection at their assigned facilities.

### 4.4. Assessment of Pilot Implementation

The goal of assessing the pilot implementation period was to document early lessons that might inform future national implementation. Overarching findings from the assessment include trainee agreement that the digital collection of RI data at the facility level was valuable and necessary for program success. Trainees also expressed that the interactive and hands-on training facilitated knowledge acquisition.

The result of the quantitative analysis (mini-data audit) revealed a discrepancy (measured as point difference) between paper-based forms at the health facility and the DHIS2 vaccination registry for the variables assessed (child enrollment, number of children vaccinated with Bacillus Calmette Guerin vaccine, and the number of children vaccinated with Diphtheria, Pertussis and Tetanus vaccine). The discrepancy was calculated by comparing the point difference between the value of these three variables (as recorded on the paper Facility Immunization Register versus the electronic DHIS2 instance). When the difference in the same variable across both sources is zero, there is no discrepancy; otherwise, a discrepancy was noted.

Table 1 presents the quantitative mini data audit comparing facility paper registers and the DHIS2-EIR instance.

For the “child enrollment” variable, record review identified 1851 (99.14%), 2854 (99.10%), and 2131 (99.35%) enrollment discrepancies between both sources for December 2023, January 2024, and February 2024, respectively. When the “number of children vaccinated with Bacillus Calmette Guerin vaccine” was assessed, record review identified a discrepancy of 318 (100%), 603 (95.41%), and 578 (99.31%) for December 2023, January 2024, and February 2024, respectively. The “number of children vaccinated with Diphtheria, Pertussis and Tetanus vaccine” variable showed a discrepancy of 845 (100%), 859 (96.09%), and 908 (99.78%). Qualitative reasons for the discrepancies included “internet issues, data not entered”; “device issues, problems with generating user ID and internet connectivity”; “same device issues persisted”; “vaccinator did not enter enrolled children in DHIS2-EIR App”; and “RI focal person struggled with device navigation”.

The qualitative analysis of the training demonstrated that 52 (34.2%) of the 152 codes (short words or phrases that summarize and categorize segments of the qualitative data collected) focused on the training experience, 44 (29.6%) on training facilitation, and 55 (24%) on participant perceptions regarding the impact of the training on job efficiency. One of the training gaps identified was that the number of days allotted for the training was too short, given that some of the participants were new learners (i.e., and new to the use of electronic devices). Some participants were in favor of transitioning to the Digital Vaccination Registry, citing its ability to reduce the time spent entering data into paper forms, ease in locating client records, and improved data preservation during collection, storage, and validation processes. However, despite these advantages, vaccinators continued to input data on paper during periods of high patient volume and later transferred the information to DHIS2-EIR on the digital devices they received during training. They also expressed the need for additional staff support, noting that the shift to DHIS2-EIR increased their workload, and therefore, may not fully replace the paper-based system in the near term. Other participants expressed a desire to maintain a paper-based system due to the potential risk of anticipated technology challenges (internet coverage, data bundle subscription, frequent malfunctioning of the devices, lack of prompt technical support to resolve issues) affecting the durability of the digital platform. They advocated gradual replacement of paper-based procedures. They asked that all necessary support infrastructure be provided.

Other issues raised by the participants were the risk of device damage, potential loss of devices, non-availability of device chargers, and risk of theft if the device was charged in a public booth.

Participants from each of the three [3] counties suggested the following improvements before the planned national rollout: the provision of a training manual to serve as a post-training reference and resource guide; the provision of adequate internet data coverage, including at the point of service delivery; and the incorporation of EIR oversight into the existing sub-national supportive supervision program to improve sustainability. Additionally, participants suggested device amendments that would improve the efficiency of the vaccination registry. The participants recommended that data fields include hospital identification numbers (in addition to the unique number generated by the device for quick service in case the mother loses the hospital card) and automatic assignment of the child’s return date or next visit. They also recommended that the mobile application provide automated vaccination status updates or progress towards the child’s required vaccinations to the vaccinator prior to the next visit.

Table 2 summarizes the main challenges and suggested improvements raised by participants during the qualitative assessment.

## 5. Implications for the Immunization Program and Conclusions

The primary objective of this assessment was to report on stakeholder engagement, system assessment, design, development, deployment, and implementation of Liberia’s Digital Vaccination Registry through the primary author’s firsthand experiences in leading this initiative. The use of a known methodology and a validated framework facilitated the success of this pilot program, particularly as the core technical team documented lessons learned that could be integrated into final vaccination registry development and its eventual national dissemination.

This work supports findings from a past study [35] that established that the successful design and deployment of digital vaccination registries is preceded by the establishment of national advisory groups, which are often responsible for driving the entire implementation process.

The needs assessment revealed a clear need for digital vaccination data management in Liberia. A fully digital RI program improves immunization data quality. Specifically, a digital system reduces the length of time between data generation and transmission to stakeholders. Issues of missing and incomplete data elements also can be resolved quickly. Errors due to manual processing and discordance with source documents can be resolved. Lastly, the system can address sub-optimal data use due to tedious monthly manual data aggregation at the health facility level. Individual digital records generated at the point of service delivery will eventually supplement the existing monthly manual aggregate reporting platform.

The high discrepancies observed between paper registers and DHIS2-EIR during the pilot should be interpreted as an early implementation challenge in the transition from paper-based workflows to digital entry at the point of service delivery. Qualitative findings suggest that intermittent internet connectivity, device and unique ID generation issues, and challenges navigating the device contributed to missed or delayed entry into the DHIS2-EIR application. In addition, vaccinators reported continuing paper documentation during periods of high patient volume and later transferring records to DHIS2-EIR, which may have contributed to discordance between sources during early rollout. In this context, discrepancy measurements serve as an implementation signal to identify barriers and guide improvements before national scale-up.

Process mapping, the definition and collection of requirements, and additional factors (aligned with relevant studies [36]) were considered to avoid exacerbating digital silos with EIR implementation. Finally, the core technical team concluded that the vaccination registry should be linked to the existing national DHIS2 electronic birth and death registration program. The unique identifying system used for birth registration should be linked to the EIR, so each child receives a single health system identification number.

DHIS2 is a digital solution that represents the ideal system described in Liberia’s definition and collection document. DHIS2 is a free and open-source software platform for collecting, analyzing, visualizing, and sharing data. It provides online and offline data entry features via a web portal, mobile application, or direct import. DHIS2 also has a well-networked global community of practice that provides technical support to its users.

Some considerations for selecting DHIS2 as the platform for the digital vaccination registry include preexisting government ownership and sustainability. Liberia’s national leadership emphasized the need to use a system that integrates into the government-managed technology stack, thus leveraging existing resources like trained personnel at all levels, existing infrastructure, and available funds from other programs using DHIS2. These considerations have been documented in previous studies [28] as contributing to successful implementation. Further, the core technical team concluded that a system with modular layered architecture that is expandable to other modules of interest (AEFI, disease surveillance, and others) could be highly beneficial to Liberia.

Following this successful pilot implementation, lessons learned and best practices will be thoroughly documented and addressed so that the planned national implementation proceeds smoothly. Finally, the use of the Collaborative Requirements Development Methodology ensured the use of tested and validated frameworks for all components.

Several elements of Liberia’s experience may be transferable to other settings: the establishment of a multi-stakeholder technical team, the use of a validated requirements development methodology, systematic process mapping and requirements definition, the selection of DHIS2 to leverage existing government infrastructure, and the training-of-trainers approach with cascaded facility trainings. At the same time, important constraints described during the pilot, particularly inconsistent internet connectivity, device logistics and maintenance, and staffing patterns during high-volume service delivery, are context-dependent and may shape feasibility and performance in other countries.

Although artificial intelligence was not evaluated in this pilot, it may be explored in future work to support data quality checks as the Digital Vaccination Registry is scaled.

## 6. Limitations

The information provided in this write-up and the findings of the different project activities should be considered with the following limitations: a limited project budget affected sample selection. The underrepresentation of other geographic areas could mean that unique phenomena related to using the digital vaccination registry in those areas were not captured. Only government facilities were included, though the MoH makes provision for routine immunization to be provided at non-profit and private health facilities. Thus, the results do not reflect the broader perspectives and experiences of the Liberian health care system.

Because facilities and counties were purposively selected by client volume and geographic accessibility, selection bias is possible and the findings may not generalize to other settings. The pilot assessment was not designed or powered to compare outcomes across counties, and county-level comparative analyses were not pre-specified.

## Figures and Tables

**Table 1:** Number of children enrolled and number of administered vaccine doses per month by county, DHIS2-EIR Pilot Assessment, Liberia, 2024. B = Facility Immunization Register; C = DHIS2-EIR instance. Percent discrepancy is calculated as (B − C)/B × 100.

Variable/Data Elements	Period	Paper Register Total (B)	DHIS2 Instance Total (C)	Absolute Difference (B − C)	% Discrepancy ((B − C)/B × 100)
Enrollment	Dec-2023	1867	16	1851	99.14
Enrollment	Jan-2024	2880	26	2854	99.10
Enrollment	Feb-2024	2145	14	2131	99.35
BCG administration	Dec-2023	318	0	318	100.00
BCG administration	Jan-2024	632	29	603	95.41
BCG administration	Feb-2024	582	4	578	99.31
DPT administration	Dec-2023	845	0	845	100.00
DPT administration	Jan-2024	894	35	859	96.09
DPT administration	Feb-2024	910	2	908	99.78

**Table 2:** Summary of participant-reported challenges and recommendations during the DHIS2-EIR assessment (qualitative findings).

Area	Participant-Reported Challenges	Participant-Suggested Improvements
Training	Insufficient training time/workshop duration; insufficient training materials (training manual); no data verification module for the data officer	Time extension; provide training manuals/handouts; include health facility demonstration/practical session; provide certification
Workflow and replacement of paper-based system	Continued paper entry during periods of high patient volume and later transfer to DHIS2-EIR; high patient load; increased workload; requires additional staff support; difficulty replacing paper-based system; gradual replacement required; expensive (including data subscriptions)	Add staff; replace paper procedures gradually; improve the system so it can support the replacement of paper-based processes
Internet/data access and connectivity	No or inadequate internet data subscription/data bundles; no or poor internet coverage; offline entry but challenges uploading/synchronizing without internet	Provide good internet coverage; provide adequate internet data coverage at the point of service delivery
System/device functionality and access	Difficult to generate automatic unique numbers/unique IDs; unresolved technical issues; no access/credentials for data officers; device not protected (no screen protector); no device charger	Create access/credentials for data officers; link DHIS2 to EIR/” direct connection”; update the application (review nationality field); provide device protector; provide chargers
Data quality practices under implementation constraints	Arbitrary data imputation when phone number or age is unknown (to complete data capture where fields cannot be left unfilled)	Improve the system to support data entry when information is unknown (as described by participants)
Device security/logistics	Risk of device damage/loss; risk of theft when charging in a public booth	Provide chargers/electricity solutions; strengthen device management support
Support, mentorship, and supervision	Limited technical support; minimal financial support for data subscriptions/recharge cards; delayed supportive supervision/mentorship (up to three months post-training); district data officer’s supervisory role not implemented; retraining required due to staff attrition	Provide technical support and mentorship; provide financial support for internet data subscriptions; incorporate EIR oversight into existing sub-national supportive supervision program
Requested system enhancements	No space for hospital number in addition to the unique number generated by the gadget; child’s return date/next visit schedule not automated; gadget does not provide vaccination status updates/progress on next visit	Add hospital number field; automate return/next visit scheduling; provide vaccination status updates/progress prompts

## Data Availability

The data presented in this study are available on request from the corresponding author due to privacy and ethical reasons.
